# The Spine pain*DETECT* questionnaire: Development and validation of a screening tool for neuropathic pain caused by spinal disorders

**DOI:** 10.1371/journal.pone.0193987

**Published:** 2018-03-21

**Authors:** Takuya Nikaido, Masahiko Sumitani, Miho Sekiguchi, Shinichi Konno

**Affiliations:** 1 Department of Orthopaedic Surgery, Fukushima Medical University School of Medicine, Fukushima, Japan; 2 Department of Pain and Palliative Medicine, The University of Tokyo Hospital, Tokyo, Japan; 3 Laboratory Animal Research Center, Fukushima Medical University School of Medicine, Fukushima, Japan; Tokyo Metropolitan Institute of Medical Science, JAPAN

## Abstract

**Objectives:**

To develop screening tools for neuropathic pain caused by spinal disorders, the Spine pain*DETECT* questionnaire (SPDQ) and its short-form version (SF-SPDQ), by modifying the Japanese version of the pain*DETECT* questionnaire (PDQ-J), and to validate these tools.

**Methods:**

Using data from patients with neuropathic pain caused by spinal disorders (NeP-SD) and patients with nociceptive pain caused by joint disorders (NocP) as controls, we devised a scoring system for the SPDQ by calculating weighting coefficients for nine PDQ-J items. Simultaneously, we selected some items for the SF-SPDQ. Next, we conducted the validation study primarily using patients with a confirmed diagnosis (a multicenter study) and general patients (a web-based survey). Sensitivity, specificity, and the area under the receiver-operating characteristic curve (AUC), along with additional positive/negative predictive values and positive/negative likelihood ratios, were calculated to assess the diagnostic utility of these tools in each population.

**Results:**

Data for 85 patients with NeP-SD and 45 patients with NocP were analyzed to develop the SPDQ/SF-SPDQ. The SPDQ had sensitivity of 78.8% and specificity of 75.6% (AUC = 0.77). The SF-SPDQ had 82.4% sensitivity and 66.7% specificity (AUC = 0.75). In the multicenter study (n = 45), both tools had diagnostic utility almost comparable with that demonstrated at development: the SPDQ had sensitivity of 83.3% and specificity of 69.2%, with the SF-SPDQ having 86.2% sensitivity and 68.8% specificity. In the web-based survey (n = 500), while the SPDQ had slightly low sensitivity (74.0%), the SF-SPDQ maintained high sensitivity (84.4%), although specificity was relatively low (61.2%).

**Conclusions:**

We developed the SPDQ and SF-SPDQ as valid screening tools for neuropathic pain caused by spinal disorders. Both have moderate utility as screening tools, with the SF-SPDQ perhaps being preferable for clinical use. However, physicians should be vigilant about possible false-positive diagnoses.

## Introduction

Neuropathic pain is defined as “pain caused by a lesion or disease of the somatosensory nervous system” [1], and is characterized as “burning,” “shooting,” or “electric shock-like” pain [2,3]. Neuropathic pain impairs sleep and increases the levels of anxiety and depression [4]. Consequently, it negatively affects patients’ health-related quality of life (HRQoL) [5].

Because a different treatment approach is required for a different type of pain [6], early identification of the involvement of neuropathic pain in patients with chronic pain is important in the appropriate management of such patients [7,8]. However, the diagnosis of neuropathic pain is difficult, especially for non-specialists in primary care settings, because various underlying mechanisms can be responsible for its development and it may coexist with other types of pain such as nociceptive or psychogenic pain [7]. Previous reports suggested that neuropathic pain is underdiagnosed and undertreated [9,10].

The pain*DETECT* questionnaire (PD-Q), one of the available screening tools for neuropathic pain [11], allows non-specialists to quickly identify potential patients with neuropathic pain [6]. The validated Japanese version of the PD-Q (PDQ-J) is also available [12]; however, it has been suggested that the PDQ-J may not have sufficient diagnostic utility in detecting neuropathic pain caused by spinal disorders [13]. A nationwide study in Japan previously reported that neuropathic pain was present in 53.3% of patients with chronic pain related to spinal disorders [14]. This high proportion highlights the need to properly identify neuropathic pain in these patients so that they can receive appropriate treatment early. In this regard, a screening tool for neuropathic pain caused by spinal disorders would be beneficial.

Our aims, therefore, were: 1) to develop a screening tool for neuropathic pain caused by spinal disorders (the Spine pain*DETECT* questionnaire, SPDQ) along with a brief, simplified version of the tool (short-form SPDQ, SF-SPDQ); and 2) to validate these tools in two different sample populations.

## Methods

### Development of the SPDQ and SF-SPDQ

We developed the SPDQ by modifying the PDQ-J to make it suitable for screening for neuropathic pain caused by spinal disorders. The PDQ-J consists of the original nine items of the PD-Q, including seven items on neuropathic pain symptoms (i.e., burning sensation, tingling sensation, pain by light touch, electric shock-like pain, pain on cold/heat stimulation, numbness, and pain by slight pressure) and two items on pain course pattern and radiating pain, respectively, with three additional numerical rating scales (NRSs) to assess the intensity of pain (i.e., current pain, most intensive pain experienced in the last 4 weeks, and average pain experienced in the last 4 weeks) [12]. A total score of the PDQ-J, calculated using the original nine items, ranges from −1 to 38 and is interpreted as indicating that the presence of a neuropathic component is unlikely (a score of ≤12), uncertain (i.e., it can be present; score 13‒18), or likely (score ≥19) [11].

In the development study of the SPDQ, we recruited patients aged 20 years or older with neuropathic pain caused by spinal disorders (NeP-SD) persisting for ≥3 months and with a pain NRS score of ≥1. The diagnosis of neuropathic pain due to spinal disorders (e.g., myelopathy, radiculopathy, and cauda equina syndrome) was confirmed by certificated orthopedic spine surgeons, on the basis of the International Association for the Study of Pain (IASP) neuropathic pain diagnostic grading system [15]. As a control group, patients with nociceptive pain caused by joint disorders (NocP) were recruited. A study questionnaire, which included the PDQ-J, was administered to patients who gave their informed consent. The aim and the contents of the study were described on the cover page of the study questionnaire. The protocol for this study was approved by the ethics committee at Fukushima Medical University School of Medicine, representing the central ethics committee, and/or ethics committees/IRBs of participating institutions. Patient recruitment was conducted at 13 hospitals in Japan from September 2011 to November 2013.

First, to devise a scoring scale for the SPDQ, we computed weighting coefficients for the nine items of the PDQ-J other than three NRSs, using canonical discriminant analysis. We then conducted receiver-operating characteristic (ROC) analyses to determine the cutoff point, which was defined as the point at which the sum of sensitivity plus specificity becomes the maximum value. In addition, to create a simplified version we selected items to be included in the SF-SPDQ using a stepwise method, and computed weighting coefficients and identified the cutoff point in the same manner as described for the SPDQ. Using the devised scoring of the SPDQ and SF-SPDQ, we calculated sensitivity, specificity, positive likelihood ratio (LR+), negative likelihood ratio (LR−), positive predictive value (PPV), negative predictive value (NPV), and the area under the ROC curve (AUC) (to discriminate patients with NeP-SD from those with NocP) for the SPDQ and SF-SPDQ.

### Validation study

To validate the SPDQ and SF-SPDQ for clinical use, we assessed the diagnostic utility of these tools using patients with a confirmed diagnosis in a multicenter study. For extended evaluation, we additionally conducted a web-based survey to assess the diagnostic utility of these tools in a more general population. Both the multicenter study and the web-based survey were approved by the ethics committee at Fukushima Medical University School of Medicine (as the central ethics committee for the multicenter study) in April 2016.

#### Multicenter study

For the multicenter study to validate the SPDQ and SF-SPDQ, we recruited patients with either NeP-SD or NocP (as a control) who were 20 years or older and had a pain NRS score of ≥3, and whose treatment was not scheduled to be changed. Patients with NeP-SD were defined as having neuropathic pain defined by the IASP [1], due to cauda equina syndrome/radiculopathy caused by lumbar spinal stenosis or due to radiculopathy caused by cervical disc herniation/lumbar disc herniation. Patients with pain suspected to be associated with nociceptive pain and patients only with low back pain or neck pain were ineligible to be included as NeP-SD patients. Patients with NocP were defined as having nociceptive pain within a month from its development or exacerbation, which was due to knee osteoarthritis/hip osteoarthritis, rheumatoid arthritis in knee/hip/limb/finger, scapulohumeral periarthritis, rotator cuff tear, or anterior and posterior cruciate ligament injury. Patients with pain suspected to be associated with neuropathic pain were ineligible to be included as NocP patients. Regardless of the type of pain (NeP-SD or NocP), patients were excluded if they had: ischemic pain or ulcer pain; pain caused by psychosocial factors or originating in mixed pain; pain caused by an act by a third party (e.g., a traffic accident); or a mental disorder, dementia, fever, or menstrual pain.

Eligible patients were identified by physicians at 10 hospitals located in or near Fukushima, Japan from May 2016 to March 2017. After physicians adequately explained the study, written informed consent was obtained from patients who agreed to participate. Physicians collected data of patients’ characteristics and asked patients to complete the first questionnaire, which included the PDQ-J and other self-report assessment measures for pain and HRQoL. No personal information was collected in the questionnaire. Among patients who visited hospitals within 2 to 4 weeks from the first questionnaire administration, patients whose symptoms had not changed since the time of the first questionnaire according to their physicians’ judgment were asked to complete the PDQ-J again (second questionnaire administration) for the assessment of repeatability of the SPDQ.

The other measures for the assessment of pain included the pain interference scale of the Brief Pain Inventory (BPI) [16,17] and the Neuropathic Pain Symptom Inventory (NPSI) [18,19]. The BPI pain interference scale assesses the extent to which pain interferes with daily activities (i.e., general activity, mood, walking ability, normal work, relations with other persons, sleep, and enjoyment of life). The scale score ranges from 0 to 10, with a larger score indicating a greater degree of interference. The NPSI is a 12-item questionnaire used to assess the severity of neuropathic pain symptoms: burning, squeezing, pressure, electric shocks, stabbing, provoked by brushing, provoked by pressure, provoked by contact with something cold, pins and needles, and tingling. A total score ranges from 0 to 100, with a larger score indicating greater severity.

The EuroQol 5 Dimension (EQ-5D) [20,21] was included for the assessment of HRQoL. The five items of the EQ-5D assess mobility, self-care, usual activities, pain/discomfort, and anxiety/depression on a 5-point scale. These five items’ scores are converted to a single index value for health status ranging from −0.025 to 1.000, with a larger value indicating better health status.

#### Web-based survey

To assess the use of the SPDQ and SF-SPDQ in a more general patient population, we recruited patients with either NeP-SD or NocP (as a control) online using an Internet research company, Rakuten Research Inc. (Tokyo, Japan), which had approximately 2.27 million registered individuals. Patients were eligible if they were 20 years or older, had pain in the upper or lower limb, and regularly received medical care for NeP-SD or NocP. Thus, patients with only low back pain or neck pain and without spinal or joint disorders were ineligible. Patients who regularly received medical care for any reason caused by an act by a third party and those who were incapable of completing a questionnaire by themselves were excluded.

An invitation with a screening questionnaire, whose purpose was to ascertain the presence of the target pain diseases (spinal disorders [i.e., lumbar spinal stenosis, lumbar spondylolisthesis, cervical spondylotic myelopathy, cervical spondylotic radiculopathy, ossification of spine ligament, or pan-spinal canal stenosis] or joint disorders [i.e., osteoarthritis, joint inflammation, or chronic rheumatoid arthritis]), was sent electronically to potential participants with spinal disorders or joint disorders. As one of several questions to check individuals’ eligibility for participation, a question to screen out patients who have both spinal disorders and joint disorders was included in the screening questionnaire. Eligible patients who had passed the screening questionnaire and agreed to participate in the survey then proceeded to the survey questionnaire, which included questions about patients’ characteristics, the PDQ-J, BPI pain interference scale, NPSI, and EQ-5D. No personal information was collected in the survey questionnaire. The survey was conducted in July to August 2016.

#### Statistical analysis in the validation study

The demographic and clinical characteristics of patients and scores of self-report assessment measures were summarized descriptively for each pain group (NeP-SD or NocP group).

The SPDQ and SF-SPDQ were scored using the scoring method devised in the development study. To assess the diagnostic utility of the SPDQ and SF-SPDQ in both the multicenter study and the web-based survey, we calculated sensitivity, specificity, LR+, LR−, PPV, NPV, and the AUC, using data obtained from the respective populations.

To assess the repeatability of the SPDQ, the agreements between the results of the first and the second administrations of the SPDQ were evaluated using Cohen’s kappa. The kappa coefficient was interpreted as follows: <0.20 as poor, 0.21–0.40 as fair, 0.41–0.60 as moderate, 0.61–0.80 as good, and 0.81–1.00 as very good [22,23].

All statistical analyses were performed using SAS release 9.4 (SAS Institute Inc., Cary, NC, USA). Data management and statistical analyses were independently conducted by Clinical Study Support, Inc. (Nagoya, Japan).

## Results

### Development of the SPDQ and SF-SPDQ

Data for 130 patients (85 patients with NeP-SD and 45 patients with NocP) were analyzed in the development study. In the NeP-SD group, mean (standard deviation, SD) age and BMI were 63.5 (14.5) years and 22.8 (3.5), and 49.4% of patients were male. In the NocP group, mean (SD) age and BMI were 60.1 (17.6) years and 23.5 (4.8), and 80.0% were male. Spinal disorders in patients with NeP-SD were mainly lumbar spinal stenosis (65 patients) and lumbar disc herniation (17 patients). Joint disorders in patients with NocP were mainly hip osteoarthritis (20 patients) and knee osteoarthritis (19 patients).

The canonical discriminant analysis yielded the weighting coefficients for nine items of the PDQ-J (Table 1). To define the SPDQ cutoff score for possible NeP-SD as ≥0, a constant term, derived as an optimized cutoff point, of −12 was defined to be added when scoring the SPDQ. To calculate the SPDQ total score, an item score of each SPDQ item, calculated by a PD-Q score multiplied by the weighting coefficient for the item, is added up for all nine SPDQ items, after which a constant value of 12 is subtracted from it.

**Table 1:** Items and scores of the SPDQ and SF-SPDQ.

	PD-Q score	Weighting coefficient	
		SPDQ	SF-SPDQ
Gradation of pain*			
Burning sensation	0–5	× (+1)	-
Tingling sensation	0–5	× (+2)	-
Pain by light touch	0–5	× (−2)	-
Electric shock-like pain	0–5	× (−4)	× (−4)
Pain on cold/heat stimulation	0–5	× (−3)	-
Numbness	0–5	× (+8)	× (+9)
Pain by slight pressure	0–5	× (+1)	-
*Pain course pattern* (select one)			
Persistent pain with slight fluctuations	0	× 0	-
Persistent pain with pain attacks	−1		
Pain attacks without pain between them	+1		
Pain attacks with pain between them	+1		
*Radiating pain*			
Yes/No	+2/0	× (+1)	-

SPDQ, Spine pain*DETECT* questionnaire; SF-SPDQ, short-form SPDQ; PD-Q, pain*DETECT* questionnaire.

*Gradation of each of the seven types of pain is answered with one of the following responses: never = 0; hardly noticed = 1; slightly = 2; moderately = 3; strongly = 4; very strongly = 5. The two questions shaded in gray are the items of the SF-SPDQ.

To calculate the SPDQ total score, an item score of each SPDQ item, calculated by a PD-Q score multiplied by the weighting coefficient for the item, is added up for all nine items, after which a constant value of 12 is subtracted from it.

To calculate the SF-SPDQ total score, an item score of each SF-SPDQ item, calculated by a PD-Q score multiplied by the weighting coefficient for the item, is added up for the two items, after which a constant value of 7 is subtracted from it.

The cutoff score for the possible spinal neuropathic pain in the SPDQ and SF-SPDQ is ≥0.

For the SF-SPDQ, two items on electric shock-like pain and numbness were selected, and weighting coefficients for these items were obtained (Table 1). Again, to define the cutoff score of the SF-SPDQ as ≥0 we set a constant term of −7, which was derived as an optimized cutoff point. The SF-SPDQ total score is calculated in the same manner as the SPDQ: an item score of each SF-SPDQ item, calculated by a PD-Q score multiplied by the weighting coefficient for the item, is added up for the two items of the SF-SPDQ, after which a constant value of 7 is subtracted from it.

For the SPDQ, the sensitivity for detecting NeP-SD was 78.8% (67/85 patients) and specificity for screening out NocP was 75.6% (34/45 patients) (Table 2). The AUC for discriminating patients with NeP-SD from those with NocP was 0.77, indicating moderate discriminant ability. For the SF-SPDQ the sensitivity was 82.4% (70/85 patients), specificity was 66.7% (30/45 patients), and AUC was 0.75. As a result, the SPDQ and SF-SPDQ, both of which have moderate utility as screening tools, were developed.

**Table 2:** Diagnostic utility of the SPDQ and SF-SPDQ at development and in two different populations in the validation study.

	n	Sensitivity	Specificity	AUC	LR+	LR-	PPV	NPV
		(%)	(%)				(%)	(%)
SPDQ								
**Development study**	**130**	**78.8**	**75.6**	**0.77**	**3.2**	**0.3**	**85.9**	**65.4**
Multicenter study	37	83.3	69.2	0.76	2.7	0.2	83.3	69.2
Web-based survey	500	74.0	72.8	0.73	2.7	0.4	73.1	73.7
SF-SPDQ								
**Development study**	**130**	**82.4**	**66.7**	**0.75**	**2.5**	**0.3**	**82.4**	**66.7**
Multicenter study	45	86.2	68.8	0.77	2.8	0.2	83.3	73.3
Web-based survey	500	84.4	61.2	0.73	2.2	0.3	68.5	79.7

SPDQ, Spine pain*DETECT* questionnaire; SF-SPDQ, short-form SPDQ; AUC, area under the receiver-operating characteristic curve; LR+, positive likelihood ratio; LR−, negative likelihood ratio; PPV, positive predictive value; NPV, negative predictive value.

### Validation study

#### Multicenter study

We obtained completed questionnaires from 29 patients with NeP-SD and 16 patients with NocP. The demographic and clinical characteristics of these patients and scores of self-report assessment measures are summarized for each group in Table 3. The mean age, BMI, and sex distribution were similar between the two groups. The mean (SD) NRS scores for current pain were 6.0 (1.8) in the NeP-SD group and 5.9 (1.6) in the NocP group. The mean NPSI scores were more than 10 points higher in the NeP-SD group than in the NocP group.

**Table 3:** Demographic and clinical characteristics of patients and scores of self-report assessment measures in the NeP-SD and NocP groups (multicenter study, n = 45).

Characteristics	NeP-SD		NocP	
	(n = 29)		(n = 16)	
Sex, male (n, %)	14 (48.3)		8 (50.0)	
Age, year	62.9 (14.1)		63.9 (15.9)	
BMI (kg/m^2^)	23.6 (3.2)		24.7 (3.5)	
Diagnosis (n, %)				
Cauda equina syndrome/radiculopathy caused by lumbar spinal stenosis	20 (69.0)		-	
Radiculopathy caused by cervical disc herniation/lumbar disc herniation	9 (31.0)		-	
Knee osteoarthritis/hip osteoarthritis	-		5 (31.3)	
Scapulohumeral periarthritis/ rotator cuff tear	-		11 (68.8)	
Disease duration, year	2.5 (3.3)		1.5 (2.6)	
Scores of measures	NeP-SD		NocP	
	n	Mean (SD)	n	Mean (SD)
Pain				
Pain NRS score (current pain)	29	6.0 (1.8)	15	5.9 (1.6)
BPI Pain Interference score	29	4.0 (2.3)	15	3.7 (2.5)
NPSI score	29	29.7 (17.2)	16	18.8 (19.9)
PDQ-J score	24	14.4 (5.1)	13	11.5 (4.9)
Health-related quality of life				
EQ-5D score	29	0.59 (0.21)	16	0.68 (0.17)

Values are mean (SD) unless otherwise indicated.

NeP-SD, neuropathic pain caused by spinal disorders; NocP, nociceptive pain caused by arthritis; BMI, body mass index; NRS, numerical rating scale; BPI, Brief Pain Inventory; NPSI, Neuropathic Pain Symptom Inventory; PDQ-J, Japanese version of the pain*DETECT* questionnaire; EQ-5D, EuroQol 5 Dimension.

Of these 45 patients, 24 patients with NeP-SD and 13 with NocP answered all items of the SPDQ; thus, the diagnostic utility of the SPDQ was analyzed using the data of these 37 patients. The mean (SD) SPDQ scores were 7.3 (9.2) in the NeP-SD group and −5.4 (8.5) in the NocP group. The SPDQ had sensitivity of 83.3% (20/24 patients) and specificity of 69.2% (9/13 patients), and the AUC was 0.76 (Table 2).

The diagnostic utility of the SF-SPDQ was analyzed using the data of all 45 patients (no missing responses). The mean (SD) SF-SPDQ scores were 9.2 (8.7) in the NeP-SD group and −3.9 (9.4) in the NocP group. The sensitivity was 86.2% (25/29 patients), the specificity was 68.8% (11/16 patients), and the AUC was 0.77.

To investigate the results of the SPDQ in each group in more detail, we examined the frequency distribution by response category for each item of the SPDQ (Table 4). For gradation of pain, more patients with NeP-SD than those with NocP reported suffering from numbness at least slightly (86.2% versus 31.3%), and no remarkable differences were observed in distributions for the remaining items. The proportions of patients with radiating pain were also similar between the two groups.

**Table 4:** Distribution by response category for each item of the SPDQ (multicenter study, n = 45).

Response category		No	Hardly Noticed	Slightly	Moderately	Strongly	Very Strongly
(PD-Q score)		(0)	(1)	(2)	(3)	(4)	(5)
*Gradation of pain*, *n (%)*							
Burning sensation	NeP-SD	4 (13.8)	8 (27.6)	8 (27.6)	6 (20.7)	1 (3.4)	2 (6.9)
	NocP	3 (18.8)	5 (31.3)	4 (25.0)	1 (6.3)	3 (18.8)	0 (0.0)
Tingling sensation	NeP-SD	2 (6.9)	2 (6.9)	6 (20.7)	11 (37.9)	5 (17.2)	3 (10.3)
	NocP	3 (18.8)	3 (18.8)	4 (25.0)	4 (25.0)	2 (12.5)	0 (0.0)
Pain by light touch	NeP-SD	6 (20.7)	15 (51.7)	7 (24.1)	1 (3.4)	0 (0.0)	0 (0.0)
	NocP	5 (31.3)	6 (37.5)	4 (25.0)	1 (6.3)	0 (0.0)	0 (0.0)
Electric shock-like pain	NeP-SD	6 (20.7)	11 (37.9)	3 (10.3)	5 (17.2)	1 (3.4)	3 (10.3)
	NocP	5 (31.3)	3 (18.8)	2 (12.5)	4 (25.0)	1 (6.3)	1 (6.3)
Pain on cold/heat stimulation^a^	NeP-SD	8 (27.6)	17 (58.6)	0 (0.0)	3 (10.3)	0 (0.0)	0 (0.0)
	NocP	9 (56.3)	7 (43.8)	0 (0.0)	0 (0.0)	0 (0.0)	0 (0.0)
Numbness	NeP-SD	1 (3.4)	3 (10.3)	9 (31.0)	11 (37.9)	4 (13.8)	1 (3.4)
	NocP	6 (37.5)	5 (31.3)	2 (12.5)	3 (18.8)	0 (0.0)	0 (0.0)
Pain by slight pressure	NeP-SD	1 (3.4)	8 (27.6)	10 (34.5)	7 (24.1)	2 (6.9)	1 (3.4)
	NocP	3 (18.8)	4 (25.0)	4 (25.0)	4 (25.0)	1 (6.3)	0 (0.0)
		NeP-SD	NocP				
		n (%)	n (%)				
*Pain course pattern*^b^							
Persistent pain with slight fluctuations		9 (31.0)	6 (37.5)				
Persistent pain with pain attacks		5 (17.2)	0 (0.0)				
Pain attacks without pain between them		5 (17.2)	8 (50.0)				
Pain attacks with pain between them		9 (31.0)	2 (12.5)				
*Radiating pain*^c^							
Yes		11 (37.9)	6 (37.5)				
No		14 (48.3)	7 (43.8)				

SPDQ, Spine pain*DETECT* questionnaire; PD-Q, pain*DETECT* questionnaire; NeP-SD, neuropathic pain caused by spinal disorders; NocP, nociceptive pain caused by arthritis.

Proportions were calculated using a denominator of 29 for the NeP-SD group and 16 for the NocP group. Shaded cells in gradation of pain indicate the response categories selected by ≥25.0% of patients in each group.

^a^Missing data for 1 NeP-SD patient.

^b^Missing data for 1 NeP-SD patient.

^c^Missing data for 4 NeP-SD and 3 NocP patients.

#### Web-based survey

A screening questionnaire was sent to 9,572 potential participants among the registered volunteers, and responses were obtained from 500 patients (250 patients for each group). The demographic and clinical characteristics of patients and scores of self-report assessment measures in each group are summarized in Table 5. The mean age and BMI were similar between the two groups. The NeP-SD group contained more males (84.8%) than the NocP group (43.6%). The mean (SD) NRS scores for current pain were similar between the two groups: 4.2 (2.4) in the NeP-SD group and 3.8 (2.3) in the NocP group.

**Table 5:** Demographic and clinical characteristics of patients and scores of self-report assessment measures in the NeP-SD and NocP groups (web-based survey, n = 500).

Characteristics	NeP-SD	NocP
	(n = 250)	(n = 250)
Sex, male (n, %)	212 (84.8)	109 (43.6)
Age, year	60.6 (10.6)	57.3 (10.5)
BMI (kg/m^2^)	24.4 (3.8)	23.6 (4.3)
Diagnosis (n, %)		
Lumbar spinal stenosis	167 (66.8)	-
Lumbar spondylolisthesis	57 (22.8)	-
Cervical spondylotic myelopathy	24 (9.6)	-
Cervical spondylotic radiculopathy	33 (13.2)	-
Ossification of spine ligament	11 (4.4)	-
Pan-spinal canal stenosis	6 (2.4)	-
Osteoarthritis	-	113 (45.2)
Joint inflammation	-	44 (17.6)
Chronic rheumatoid arthritis	-	110 (44.0)
Disease duration^a^, year	8.3 (8.4)	11.4 (12.0)
Pain		
Pain NRS score (current pain)	4.2 (2.4)	3.8 (2.3)
BPI Pain Interference scale score	2.7 (2.5)	2.3 (2.2)
NPSI score	21.1 (18.3)	16.8 (15.7)
PDQ-J score	11.2 (5.7)	9.1 (5.2)
Health-related quality of life		
EQ-5D score	0.76 (0.17)	0.78 (0.16)

Values are mean (SD) unless otherwise indicated.

NeP-SD, neuropathic pain caused by spinal disorders; NocP, nociceptive pain caused by arthritis; BMI, body mass index; NRS, numerical rating scale; BPI, Brief Pain Inventory; NPSI, Neuropathic Pain Symptom Inventory; PDQ-J, Japanese version of the pain*DETECT* questionnaire; EQ-5D, EuroQol 5 Dimension.

^a^For patients who were diagnosed with two or more diseases, disease duration was calculated for the disease of longest duration.

The mean (SD) SPDQ scores were 6.7 (10.4) in the NeP-SD group and −5.4 (9.0) in the NocP group. The SPDQ had sensitivity of 74.0% (185/250 patients) and specificity of 72.8% (182/250 patients), and the AUC was 0.73 (Table 2). The mean (SD) SF-SPDQ scores were 10.7 (10.0) in the NeP-SD group and −1.6 (9.2) in the NocP group. The sensitivity was 84.4% (211/250 patients), the specificity was 61.2% (153/250 patients), and the AUC was 0.73.

Table 6 shows the frequency distribution by response category for each item of the SPDQ. More than 80% of the NeP-SD group reported suffering from numbness, at least slightly, whereas more than 60% of the NocP group reported having had no, or hardly noticing, numbness. For pain course pattern and radiating pain, the response distribution was similar between the two groups.

**Table 6:** Distribution by response category for each item of the SPDQ (web-based survey, n = 500).

Response category		No	Hardly Noticed	Slightly	Moderately	Strongly	Very Strongly
(PD-Q score)		(0)	(1)	(2)	(3)	(4)	(5)
*Gradation of pain*, *n (%)*							
Burning sensation	NeP-SD	70 (28.0)	95 (38.0)	39 (15.6)	32 (12.8)	12 (4.8)	2 (0.8)
	NocP	108 (43.2)	94 (37.6)	26 (10.4)	18 (7.2)	4 (1.6)	0 (0.0)
Tingling sensation	NeP-SD	31 (12.4)	63 (25.2)	64 (25.6)	66 (26.4)	21 (8.4)	5 (2.0)
	NocP	66 (26.4)	74 (29.6)	66 (26.4)	36 (14.4)	6 (2.4)	2 (0.8)
Pain by light touch	NeP-SD	93 (37.2)	117 (46.8)	23 (9.2)	14 (5.6)	2 (0.8)	1 (0.4)
	NocP	93 (37.2)	111 (44.4)	32 (12.8)	10 (4.0)	3 (1.2)	1 (0.4)
Electric shock-like pain	NeP-SD	68 (27.2)	79 (31.6)	64 (25.6)	23 (9.2)	10 (4.0)	6 (2.4)
	NocP	73 (29.2)	70 (28.0)	53 (21.2)	37 (14.8)	16 (6.4)	1 (0.4)
Pain on cold/heat stimulation	NeP-SD	118 (47.2)	103 (41.2)	18 (7.2)	10 (4.0)	1 (0.4)	0 (0.0)
	NocP	125 (50.0)	96 (38.4)	24 (9.6)	5 (2.0)	0 (0.0)	0 (0.0)
Numbness	NeP-SD	9 (3.6)	37 (14.8)	63 (25.2)	94 (37.6)	36 (14.4)	11 (4.4)
	NocP	72 (28.8)	89 (35.6)	55 (22.0)	28 (11.2)	6 (2.4)	0 (0.0)
Pain by slight pressure	NeP-SD	35 (14.0)	103 (41.2)	59 (23.6)	41 (16.4)	8 (3.2)	4 (1.6)
	NocP	29 (11.6)	68 (27.2)	72 (28.8)	62 (24.8)	13 (5.2)	6 (2.4)
		NeP-SD	NocP				
		n (%)	n (%)				
*Pain course pattern*							
Persistent pain with slight fluctuations		112 (44.8)	100 (40.0)				
Persistent pain with pain attacks		40 (16.0)	51 (20.4)				
Pain attacks without pain between them		55 (22.0)	70 (28.0)				
Pain attacks with pain between them		43 (17.2)	29 (11.6)				
*Radiating pain*							
Yes		66 (26.4)	69 (27.6)				
No		184 (73.6)	181 (72.4)				

SPDQ, Spine pain*DETECT* questionnaire; PD-Q, pain*DETECT* questionnaire; NeP-SD, neuropathic pain caused by spinal disorders; NocP, nociceptive pain caused by arthritis.

Proportions were calculated using a denominator of 250 for each group. Shaded cells in gradation of pain indicate the response categories selected by ≥25.0% of patients in each group.

#### Repeatability

We calculated Cohen’s kappa coefficients to assess the agreements between the results of the first and the second questionnaire administrations, using the data of 21 patients with NeP-SD and 8 patients with NocP who completed the SPDQ twice in the multicenter study. Similarly, the kappa coefficients for the SF-SPDQ were calculated using the data of 25 patients with NeP-SD and 11 patients with NocP who completed the SF-SPDQ twice. The kappa coefficient was 0.5 for both the SPDQ and the SF-SPDQ, indicating that the agreement was moderate.

## Discussion

We developed a screening tool to help detect patients with neuropathic pain caused by spinal disorders and also a brief, simplified version, both of which had moderate utility as screening tools. Subsequently we assessed the diagnostic utility of these tools using two different populations, and the multicenter study using patients with a confirmed diagnosis showed that both the SPDQ and SF-SPDQ had moderate diagnostic utility also in this population. Furthermore, the SF-SPDQ maintained reasonably high sensitivity in our extended evaluation using general patients, supporting its preferable use in clinical settings.

In the multicenter study, the SPDQ had sensitivity and specificity almost comparable with those demonstrated in the development study (sensitivity, 83.3% versus 78.8%; specificity, 69.2% versus 75.6%). The SF-SPDQ had sensitivity and specificity that were close to those at development (sensitivity, 86.2% versus 82.4%; specificity, 68.8% versus 66.7%). The PPV and NPV of each tool were also similar to those in the development study, where the prevalence of the target pain was almost the same as that in this population. These comparable results support the idea that both the SPDQ and SF-SPDQ may have equivalent diagnostic utility in similar clinical settings (e.g., orthopedic surgery department). Although the PPV of 83.3% for both tools implies that positive results are likely to be reliable in this population, the relatively low specificity still suggests that physicians should be mindful of the potential false-positive diagnoses. The LR+ of <3 was not high enough: LR+ of ≥10 is considered to indicate that a positive test result would be good at “ruling in” the disease [24], which also emphasizes the importance of further examination of patients evaluated as positive to appropriately rule out patients without neuropathic pain.

In the web-based survey, we collaterally evaluated the use of these tools in a more general patient population. Compared with the results of the multicenter study, the SPDQ had low sensitivity (74.0%) in this population. This may be attributed to the mild pain conditions, as shown by the low mean scores for pain NRS and the NPSI. Indeed, many patients with NeP-SD reported having had no pain, or hardly noticed their pain, for most symptoms (see Table 6); as a result, patients had an SPDQ score of <0, resulting in false-negative diagnoses. By contrast, the SF-SPDQ maintained reasonably high sensitivity (84.4%) in this population, although the specificity was low (61.2%). One explanation for the low specificity may be that these tools do not include items to assess factors associated with the development of symptoms of spinal disease, i.e., spinal dynamic factors and postural factors. Thus, despite the lack of these factors in patients, some with NocP might have been evaluated as NeP-SD by these tools when they had neuropathic pain-like symptoms. Another possible reason, especially in this web-based survey, may lie in the fact that because neuropathic pain can also exist in a certain proportion of patients with arthritis or joint diseases [25–27], some patients with NocP included in this survey based on self-reported diseases might have had neuropathic pain as well as nociceptive pain. However, considering that screening is the purpose of these tools, the SF-SPDQ, which is shorter and yet consistently has high sensitivity, may be preferable to the SPDQ for clinical use.

The examination of the response distribution to each SPDQ item revealed that more patients with NeP-SD reported numbness in comparison with control patients. This was not surprising because the item for numbness is weighted the most in the SPDQ and SF-SPDQ. However, the fact that conspicuous distributions were observed in both populations may suggest that numbness can be one clue to the probable presence of neuropathic components in Japanese patients with pain, although it does not necessarily deny other types of pain such as nociceptive pain. For the remaining items, the response distribution did not greatly differ between the two pain groups, even for the item for electric shock-like pain, which is weighted the second most in the SPDQ and SF-SPDQ. As discussed above, these similarities—more precisely, less frequent manifestation of these pain symptoms—probably reflect the mild conditions of patients. Because the SPDQ is based on patients’ description of pain symptoms, it would be more suitable for patients with pain of a certain level of severity.

The item for electric shock-like pain is given the largest negative coefficient in both tools, meaning that the possibility of neuropathic pain decreases if patients have stronger electric shock-like pain. Considering that the term “electric shock-like pain” is one descriptor of neuropathic pain [2], this seems contradictory. However, this term is also used by patients with osteoarthritis to describe their pain symptoms [28,29]. Thus, although we cannot deny the presence of neuropathic pain, “electric shock-like pain” reported by patients with NocP would also reflect the sudden, extremely acute pain experienced in patients with osteoarthritis rather than that suggestive of neuropathic pain.

For repeatability, the agreement was moderate for both the SPDQ and SF-SPDQ. One possible reason for this may be attributed to changes in patients’ symptoms, although patients included in the second questionnaire administration were supposed to be those without changes in symptoms as judged by physicians. Because patients were required to describe their symptoms in detail in the SPDQ, rather than their overall condition, possible changes in patients’ perception of their symptoms might have resulted in different responses to the SPDQ. One might also think that it would reflect the fluctuation of symptoms. However, because the mean disease duration was relatively long (2.5 years in the NeP-SD group and 1.5 years in the NocP group), it is more likely that their diseases were formed by the time of the questionnaire administration, which was confirmed by the physicians’ judgment of stable symptom conditions. Thus, we consider that the possible changes in responses probably reflected a more subjective level of change rather than the fluctuation of symptoms.

There are some limitations to this study. First, because the SPDQ and SF-SPDQ were developed using a statistical model, the resulting tools were highly dependent on the characteristics of the sample population. Therefore we conducted the validation study using patients with a confirmed diagnosis, and the results showed that both tools had equivalent diagnostic utility also in a different population. Second, patients in our multicenter study were recruited from a limited area of Japan, which may limit generalization to patients across the nation. However, the results were comparable with those of the development study of the SPDQ, for which patients were enrolled from various regions of Japan. The generalizability of the results of our web-based survey may also be limited because some groups of patients may have been under-represented. For example, patients suffering severe pain might not have been able to or willing to participate, which may have resulted in the inclusion of patients with less severe symptoms. Third, recruiting a feasible number of patients for our multicenter study resulted in a small sample size, meaning that one patient’s data largely influenced the results of the study. In addition, our validation study relied on patients’ self-reporting in the questionnaires, which may have resulted in misclassification. In particular, inclusion of patients in our web-based survey according to their self-reported diseases may have resulted in improper inclusion or misclassification of patients, despite our best efforts to screen out inappropriate patients by using a screening questionnaire. Given these limitations, the results of this study need to be interpreted with caution.

### Conclusion

We developed a valid screening tool for neuropathic pain caused by spinal disorders, the SPDQ, and a simplified version, the SF-SPDQ. Both the SPDQ and SF-SPDQ demonstrated moderate diagnostic utility in patients attending the orthopedic department, but the SF-SPDQ may be more suitable for use in clinical settings because although it is a 2-item short tool it consistently demonstrated high sensitivity in two different populations. However, physicians should be aware of the possibility of false-positive diagnoses. Additional examinations of patients evaluated as positive may help to more accurately detect patients with neuropathic pain caused by spinal disorders.
